# Lane Mark Detection with Pre-Aligned Spatial-Temporal Attention

**DOI:** 10.3390/s22030794

**Published:** 2022-01-21

**Authors:** Yiman Chen, Zhiyu Xiang

**Affiliations:** 1 College of Information Science & Electronic Engineering, Zhejiang University, Hangzhou 310027, China; chenyiman@zju.edu.cn; 2 Zhejiang Provincial Key Laboratory of Information Processing, Communication and Networking, Zhejiang University, Hangzhou 310027, China

**Keywords:** lane mark detection, pre-aligned multiple frames, Spatial-Temporal Attention

## Abstract

Lane mark detection plays an important role in autonomous driving under structural environments. Many deep learning-based lane mark detection methods have been put forward in recent years. However, most of current methods limit their solutions within one single image and do not make use of the de facto successive image input during the driving scene, which may lead to inferior performance in some challenging scenarios such as occlusion, shadows, and lane mark degradation. To address the issue, we propose a novel lane mark detection network which takes pre-aligned multiple successive frames as inputs to produce more stable predictions. A Spatial-Temporal Attention Module (STAM) is designed in the network to adaptively aggregate the feature information of history frames to the current frame. Various structure of the STAM is also studied to ensure the best performance. Experiments on Tusimple and ApolloScape datasets show that our method can effectively improve lane mark detection and achieve state-of-the-art performance.

## 1. Introduction

With the rapid development of autonomous driving technology, lane mark detection have made great progress in recent years. Accurate and robust lane mark detection is necessary to ensure the safety of autonomous navigation in terms of its capability to provide reliable route guidance and proper positioning for the vehicle. However, lane mark detection under complex scenes and various light conditions still remains a challenge.

Traditional methods for lane mark detection usually involve several basic procedures, including image pre-processing, feature extraction, and detection by fitting [1,2,3]. They heavily rely on highly-specialized and hand-crafted feature extraction [4,5,6]. Thanks to the emergence of deep neural network and large-scale datasets, deep learning methods have significantly improved the performance of lane mark detection. Liu et al. [7] proposed a style-transfer-based data enhancement method, using Generative Adversarial Networks (GANs) to solve the problem of lane detection in low-light conditions. RESA [8] shifted sliced feature map recurrently in vertical and horizontal directions to aggregate global information, which helps to conjecture lane marks with weak appearance coherences. To better infer lane mark positions under occlusion conditions, LaneATT [9] utilized an effective anchor-based attention mechanism to aggregate global information. However, most of the methods focus on detecting lane marks in a single image. Under complex environments, the appearance of lane marks can be frequently degraded by severe stains, heavy shadows, or serious occlusion, which can result in incomplete or even incorrect predictions for these single image-based methods. In practice, the image sequence acquired by the vehicle are continuous and there are large overlaps between adjacent frames, therefore the position of lane marks in neighboring frames are highly correlated. In other words, lane marks that cannot be precisely detected in a current single frame is able to be inferred from the information of former frames. This motivates us to investigate lane mark detection with multiple frames as input and explore the inherent spatial-temporal information within the sequence.

In this work, a novel method using multiple frames for improving lane mark detection is proposed. To maximize the enhancement for the features of a current key frame, we first perform multi-frame pre-alignment. While the camera calibration in [10] establishes one-to-one correspondence between the image plane and the ground, we project each history frame to the current key frame with the road areas aligned in the image plane. Moreover, to further aggregate spatial-temporal information, we propose an effective Spatial-Temporal Attention Module (STAM) and insert it into an encoder-decoder-based instance segmentation network. Taken multiple continuous images as inputs, sequential features of all input frames are extracted by the shared CNN encoders and then fed into the STAM. A two-branch decoder is adopted to reconstruct the aggregated information and predict lane marks of the current key frame. With richer information from continuous images, the proposed method is able to greatly improve lane mark predictions on challenging scenarios and achieve state-of-the-art performance.

The main contributions of this paper can be summarized as:

- We regard lane mark detection as a time-series issue and propose to detect lane marks from successive pre-aligned multiple images. The frames are pre-aligned according to the ground plane before feeding to the network. By exploring the spatial-temporal information hidden in the multiple frames, the negative influence from complex scenarios like shadow, lane mark degradation, and vehicle occlusion could be largely mitigated;
- A novel Spatial-Temporal Attention Module (STAM) is proposed and embedded in the encoder-decoder backbone. The module enhances the features of current frame by attentively aggregating spatial-temporal information from history frames. Various structures of the STAM and their performance are also studied;
- Our network is implemented end-to-end and evaluated on two large-scale datasets: Tusimple and ApolloScape. Comprehensive experiments and ablation studies verified that the proposed model is effective and can achieve state-of-the-arts performance.

## 2. Related Work

Lane mark detection has been intensively researched in recent years. These methods can be roughly classified into traditional and deep learning approaches.

**Traditional** methods. Before the advent of deep learning, conventional solutions for lane mark detection often depend on hand-crafted features such as edge, color, and texture to identify lane segments [4,5,6]. Then, Hough transform [11] or curve fitting [12] is often adopted to eliminate outliers and form the final lane marks. Apart from geometric modeling, some methods formulate lane mark detection with energy minimization algorithms [13]. By defining unary/dual potentials and building an optimal association of multiple lane marks, Conditional Random Field (CRF) can be used to detect lane marks. For lane mark detection in successive frames, the particle or Kalman filter is widely used [14,15,16]. The particle filter is able to track multiple lanes. The Kalman filter helps to locate positions and estimate lane curvature with state vectors. However, the performance of the above methods would be easily mortified by complex environments and illumination variance.

**Deep-learning-based methods.** In recent years, many deep-learning-based methods on lane mark detection have been proposed. According to the representations of lane, the existing methods can be divided into four categories: Segmentation-based [8,17,18,19,20], anchor-based [9,21,22], row-wise detection-based [23,24,25], and parametric regression methods [26,27]. Segmentation-based methods are the most popular and have an impressive performance. SCNN [18] employed slice-wise convolution in a segmentation module, passing a message from different directions to capture spatial continuity. EL-GAN [19] and SAD [20] respectively adopted GAN and knowledge distillation to improve lane mark segmentation. Despite their advantages, most segmentation-based methods are limited to detecting lane marks with a pre-defined number. Anchor-based methods focus on specifying the lane mark shape by regressing the position offsets relative to the predefined anchors. PointLaneNet [21] used point anchors to directly obtain the coordinates of lane mark points. Line-CNN [22] put forward a novel Line Proposal Unit (LPU) in terms of discrete direction classification and relative coordinate regression. LaneATT [20] extracted anchor-based features and utilized an attention mechanism. However, a fixed anchor shape would be inflexible to describe lane marks with a high degrees of freedom. Row-wise detection methods predict the most probable location of lane marks from row to row. Fast-Draw [23] introduced a learning-based approach to decode the lane mark structure without post-processing. UFSA [24] proposed a lightweight row-based selecting scheme in global image features, resulting in a high speed algorithm. E2E-LMD [25] predicted lane mark vertexes in an end-to-end manner. Parametric regression methods directly output parametric representations of lane marks. PolyLaneNet [26] learned to regress the lane mark polynomial curve equation. LSTR [27] formulated the lane mark shape model based on road structures and camera pose, using a transformer to capture a richer context.

In contrast to the above single-frame based methods, a few approaches consider the lane mark detection as a time-series problem. Zou et al. [28] proposed a hybrid architecture that seamlessly integrates the CNN (Convolutional Neural Network) [29] and RNN (Recurrent Neural Network) [30] to detect lane marks. Zhang et al. [31] added double Convolutional Gated Recurrent Units (ConvGRUs) into an encoder-decoder CNN. However, they only consider the lane detection as a two-class segmentation problem and did not provide instance segmentation for each lane. Moreover, in complex scenes such as lane occlusion by dynamic vehicles, they are also prone to produce erroneous false positive predictions. Our method takes instance-level discrimination into account and perform multi-frame pre-alignment before feeding them into the network. Instead of using RNN or any variants of RNN, we propose STAM to aggregate the spatial-temporal information to better deal with the challenging scenarios.

## 3. Proposed Methods

**Figure 1:**
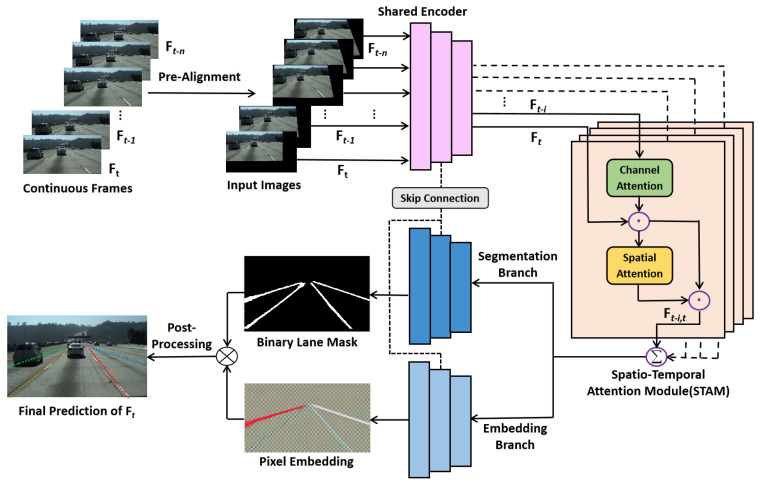
Overview of the proposed method. Multiple pre-aligned consecutive frames are firstly sent to the shared encoder. Then, the features of current key frame $F_{t}$ are enhanced by attentively aggregating spatial-temporal information from history frames $F_{t-i}$. After that, the two-branch decoder produces a binary lane mask and an N-dimensional embeddings per lane pixel. At last, the post-processing is applied to gain the final predictions.

As detecting lane marks from individual images suffers from challenging situations such as heavy shadow, serious occlusion, and severe lane mark damage, we focus on lane mark detection under continuous driving scenes. Among consecutive images, lane marks in adjacent frames are inherently correlative. An overview of our proposed method is illustrated in Figure 1. The encoder-decoder network takes multiple pre-aligned consecutive frames as inputs and predicts lane marks on the current key frame $F_{t}$ in an instance segmentation manner. Sequential encoded features are aggregated by the proposed Spatial-Temporal Attention Module (STAM), followed by a decoder to receive the fusion feature. The decoder consists of two branches: The segmentation branch generates a binary lane mask with two classes (lane or background), the embedding branch is trained to disentangles the segmented lane pixels into different lane instances. Finally, predicted lane mark points are obtained by the post-processing.

### 3.1. Multi-Frame Pre-Alignment

**Figure 2:**
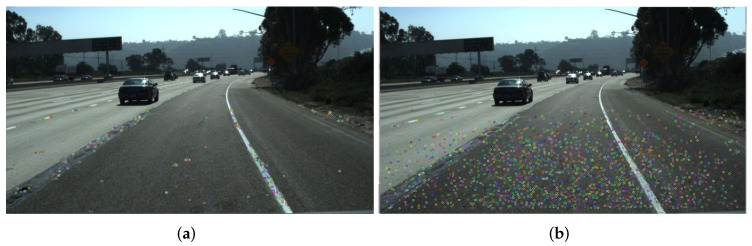
The comparison of feature points extraction between using (**a**) Opencv and (**b**) our method.

To adequately enhance the features of current key frame and avoid introducing confusion among different images, alignment of multiple frames is necessary. This section will explain the procedures of multi-frame pre-alignment. The lane marks we are interested in are all on the ground plane. Assuming the ground area ahead of the vehicle is locally planar, a 2D homographic transformation can be set up for the ground area between neighboring frames. We assume the image rows under the predefined vanishing lines are the ground area and compute the homographic transformation by feature point matching. However, in practice the ground is often composed of a weak texture area, which means insufficient feature points could be extract, as shown in Figure 2a. We solve this problem by extracting evenly distributed ORB (Oriented FAST and Rotated BRIEF) [32] feature points. Specifically, we divide the area into 30×30 grids and detect FAST (Features from Accelerated Segment Test) [33] corners with Non-Maximum Suppression (NMS). If insufficient corners are found in the grids, the detector threshold is adjusted adaptively. After a certain number of FAST corners are extracted, the corresponding rotated BRIEF (Binary Robust Independent Elementary Features) [34] descriptors are computed. Then, we employ QuadTree to administrate the features, making them evenly distributed and having them meet the quantity requirements simultaneously. As shown in Figure 2, our method for feature points extraction works better than simply using the Opencv library.

**Figure 3:**
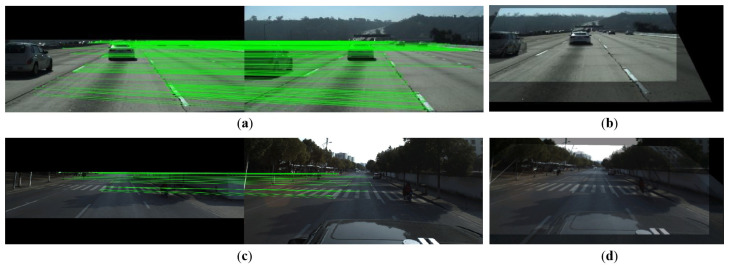
Illustration of image pre-alignment for consecutive two frames. The top row shows the example from the Tusimple [36] dataset and the bottom is from the ApolloScape [37] dataset. (**a**,**c**) represent the procedure of feature points matching, (**b**,**d**) indicate the results of alignment.

After feature extraction, we conduct feature point matching for each pair of images. RANSAC (RANdom SAmple Consensus) [35] is performed to compute the homographic matrix between the previous frame and current frame. Then we can warp the previous frames to the current frame, realizing the multi-frame pre-alignment. The visualization examples for the procedure of feature points matching and inter-frame warpping are presented in Figure 3, where we can observe that the lane marks of two frames are exactly aligned with each other. Note that all the aligned images should be padded to the same resolution before input to the network.

### 3.2. Instance Segmentation Network

For instance, segmentation of lanes, an encoder-decoder architecture is employed, which uses VGG16-based FCN [38] as the backbone. The encoder CNN extracts the sequential features for all input frames. The decoder CNN consists of a binary segmentation branch and a pixel embedding branch. The binary segmentation branch decides the class of background or lane mark, while the embedding branch further disentangles the segmented lane mark pixels into different lane instances. The binary segmentation branch is trained by the standard cross-entropy loss function, using bounded inverse class weighting [39] to handle classes (lane/background) unbalance.

The instance embedding branch is trained to assign a lane ID to each lane pixel so that the pixel embeddings belonging to the same lane are pulled closer, whereas those belonging to different lanes are pushed away. In this way, the pixel embeddings of the same lane will cluster together to generate unique instance. The clustering loss function [40] for the instance embedding branch is: $L=\alpha L_{var}+\beta L_{dist}+\gamma L_{reg}$, where α, β, and γ are weighting coefficients, and the three loss items are:

(1) $$\left\{\begin{array}{c} L_{var}=\frac{1}{C}\sum_{c=1}^{C}\frac{1}{N_{c}}\sum_{i=1}^{N_{c}}{\left[∥\mu_{c}-x_{i}∥-\delta_{v}\right]}_{+}^{2}\\ L_{dist}=\frac{1}{C(C-1)}\sum_{c_{A}=1}^{C}\sum_{c_{B}=1{,}_{c_{A}\neq c_{B}}}^{C}{\left[\delta_{d}-∥\mu_{c_{A}}-\mu_{c_{B}}∥\right]}_{+}^{2}\\ L_{reg}=\frac{1}{C}\sum_{c=1}^{C}∥\mu_{c}∥. \end{array}\right.$$

In Equation (1), C represents the number of lane mark clusters, $N_{c}$ denotes the number of elements in cluster c, $x_{i}$ is a pixel embedding, µc is the mean embedding of cluster c, $\delta_{v}$ and $\delta_{d}$ are thresholds, and $∥\cdot ∥$ indicates the $L_{2}$ distance, ${\left[x\right]}_{+}=max\left(0,x\right)$. The variance term ($L_{var}$) applies a pull force on each pixel embedding towards the mean embedding of a cluster, which is only active when the embedding is farther than $\delta_{v}$ from its cluster center. The distance term ($L_{dist}$) serves to push the cluster centers away from each other. The push force is only effective when the distance between these centers is closer than $\delta_{d}$.

### 3.3. Spatio-Temporal Attention Module

**Figure 4:**
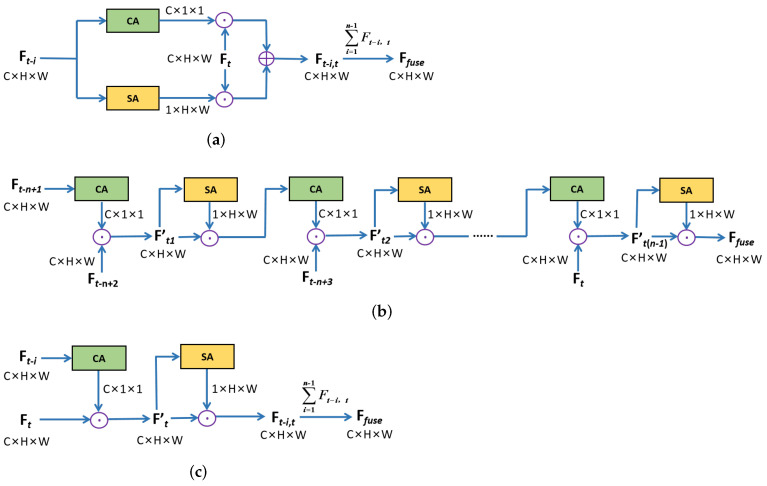
Three configuration modes for Spatial-Temporal Attention Module (STAM). (**a**) Parallel mode. (**b**) Serial mode. (**c**) The mixed mode.

To effectively fuse the encoded features from a multi-frame, we propose a Spatial-Temporal Attention Module (STAM) and insert it between the encoder and decoder. The module extracts Channel Attention (CA) and Spatial Attention (SA) from previous frames and applies them on the current frame for feature aggregation. According to the different connection manner of the two attentions and their acting target frames, STAM can be constructed by three modes, i.e., parallel, serial, and mixed mode, as shown in Figure 4. We assume that the size of the input tensor is C×H×W, where *C*, *H*, *W* are the number of elements along the channel, height, and width dimension, respectively.

In parallel mode, CA and SA respectively take the feature of previous frame $F_{t-i}$ as input to generate temporal and spatial attention map in a parallel manner. Then, the two attention maps are multiplied to the feature of current frame $F_{t}$ followed by element-wise addition to produce the temporary fused feature $F_{t-i,t}$. The temporary fused features generated by all of the previous frames are then further aggregated by $F_{\mathrm{fuse}}=\sum_{i=1}^{n-1}F_{t-i,t}$, where n indicates the number of input frames. The second attention fusion way is to successively aggregate the history frame in a serial mode. As shown in Figure 4b, the feature of history frame $F_{t-i}$ is firstly fed to CA, after applying the resulting attention to $F_{t-i+1}$, the intermediate result is further input to SA to generate a two-frame aggregated feature $F_{t-i,t-i+1}$. Then the result is regarded as input of the CA of the next frame and the aggregation starts until the current frame is processed. Note that the order of CA and SA is exchangeable. The third way is the mixed mode, where the attention is applied between each pair of $F_{t-i}$ and $F_{t}$ serially, while the final aggregation is implemented by summation just like in the parallel mode. The detailed experimental studies for the different modes are conducted in Section 4.2.

**Figure 5:**
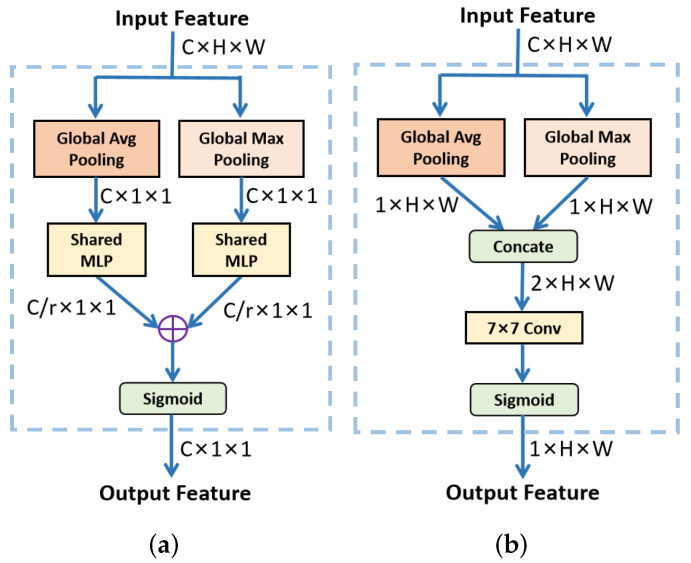
The detailed architectures of CA and SA in STAM. (**a**) Channel Attention (CA). (**b**) Spatial Attention (SA).

The specific architectures of CA and SA in STAM are illustrated in Figure 5. As shown in Figure 5a, the CA employs global average-pooling and global max-pooling to integrate spatial information of input features. After being processed by a shared Multi-layer Perception (MLP), the feature vectors are aggregated by element-wise summation to generate a channel attention $M_{C}\left(i\right)$:

(2) $$M_{C}\left(i\right)=\sigma \left(MLP\left(MaxPool\left(F_{t-i}\right)\right)+MLP\left(AvgPool\left(F_{t-i}\right)\right)\right),i=1,2,\cdots ,n-1$$

where σ indicates the sigmoid function and n is the number of continuous frames. For SA, average-pooling and max-pooling operations are applied along the channel axis. The pooled features are concatenated and transmitted to a standard convolution layer, producing a spatial attention map $M_{S}\left(i\right)$ as:

(3) $$M_{S}\left(i\right)=\sigma \left(f^{7\times 7}\left(\left[MaxPool\left(F_{i}^{{}^{\prime }}\right);AvgPool\left(F_{i}^{{}^{\prime }}\right)\right]\right)\right),i=1,2,\cdots ,n-1$$

where $f^{7\times 7}$ denotes a convolution operation with a 7×7 filter size.

### 3.4. Post-Processing

As we regard lane mark detection as an instance segmentation problem, the inference of the arbitrary number of lane marks is allowed and lane changes can be handled. Since the pixel embedding of the same lane mark has been assigned by the network, DBSCAN (Density-Based Spatial Clustering of Applications with Noise) [41] algorithm is applied to determine the clustering category and form the unique lane mark instance. To get the final detection result, precise coordinates of the lane mark have to be distilled from the candidate areas. Here, we first sample lane points along the y axis for every 10 pixels, then perform curve fitting for a simpler description of lane marks and filtering out the outliers.

## 4. Experiments

### 4.1. Experimental Setting

#### 4.1.1. Datasets

To extensively evaluate the proposed method, we conduct experiments on two datasets: Tusimple and ApolloScape. Both of the datasets provide image sequences for training and testing.

**Tusimple.** TuSimple [36] is widely used in the existing works of lane mark detection. It is collected on highway roads under nice weather conditions at different daytimes. The images have a resolution of 1280×720 and contain 2–5 lanes for detection. The dataset consists of 3626 and 2782 image sequences for training and testing, respectively. Each sequence comprises 20 continuous frames with only the last frame annotated by sampling points. To construct the ground-truth binary and instance segmentation map for training, we connect all of the annotated points together to form an intact curve per lane.

**ApolloScape.** ApolloScape [37] is a large scale dataset that is provided by Baidu corporation. It contains seven different tasks for autonomous driving including lane segmentation. For this task, a diverse set of stereo video sequences are recorded in urban traffic scenarios with high quality pixel-level annotations. The resolution of images in ApolloScape is 3384×2710. Since the Apolloscape lane dataset only provides pixel-level semantic annotations without instance-level discrimination, and we only focus on detecting lane marks rather than recognizing all of the 35 categories in the dataset. We selected 5519 frames and annotated them with sampling points interpolated by cubic spline. For each training image, the previous 4 frames are provided for input without labeling. The split dataset is divided into 3317 frames for training, 608 for validation, and 1595 for testing.

#### 4.1.2. Implementation Details

Our model is implemented on Tensorflow [42] with GPU GTX 1080Ti. The network is trained with an embedding dimension of 4 with $\delta_{v}=0.5$, $\delta_{d}=3$, α=1, β=1, γ=0.001. All images are rescaled to 512×256 with nearest interpolation. During the training process, we employ a SGD (Stochastic Gradient Descent) [43] optimizer with a base learning rate of $5\times 10^{-3}$, momentum of 0.9 and batch size of 4. A poly learning rate policy is used with power 0.9 and maximal iteration 100 K. We also applied data augmentation including random cropping, random horizontal flipping, and color augmentations.

#### 4.1.3. Evaluation Criteria

For ablation studies and comparisons with other lane mark detection methods, different metrics are adopted to evaluate the results on each particular dataset.

**Tusimple.** Here, we follow the official evaluation criteria [36]. The predicted lanes are sampled by points with fixed intervals along the y axis. Predicted points whose distance to the ground truth is less than 20 pixels are regarded as the correct points. The accuracy is calculated as:

(4) $$acc=\sum_{im}\frac{C_{im}}{S_{im}}$$

where $C_{im}$ is the number of correct points and $S_{im}$ is the total number of lane points in the image. Lane marks with an accuracy greater than 85% are considered as True Positive (TP), otherwise False Positive (FP) or False Negative (FN). The F1-measure is taken as the primary evaluation metric, which is computed as:

(5) $$F1=2\times \frac{Precision\times Recall}{Precision+Recall}$$

where $Precision=\frac{TP}{TP+FP}$, $Recall=\frac{TP}{TP+FN}$.

**ApolloScape.** While Tusimple uses distance metric, evaluation on ApolloScape refers to the area-metric used in the CULane dataset [18]. Each lane marking is viewed as a 30-pixel-width line connecting the sampled lane points. We calculate the IoU (Intersection-over-Union) [44] between the ground-truth and prediction. In lane-wise fashion, predicted lane instance is counted as True Positive (TP) when its IoU is higher than a certain threshold. We consider 0.3 and 0.5 thresholds corresponding to loose and strict evaluations for the experiments on ApolloScape. The F1 score is also treated as the major evaluation metric, which is defined as mentioned earlier.

### 4.2. Ablation Study

To verify our method, we will make comprehensive ablation studies on the Tusimple dataset carried out in this section.

**Effects of multi-frames.** Firstly, we investigate the effectiveness of aggregating information from multiple frames. As shown in Table 1, compared with a single frame baseline, using multiple frames does help to increase the accuracy and reduce the wrong predictions. It can be explained that multi-frame fusion brings richer information and enhances the feature of current frame, which helps to improve the performance. Note that in the 2nd row of Table 1, we also list the results of baseline equipped with the proposed STAM for comparison. The results show that employing 4 frames can obtain the best performance with a F1 score 3.51% higher than the original single-frame baseline. Although adopting 5 frames has a comparable accuracy with 4 frames, we empirically use 4 frames in our method by considering the trade-off between the computing cost and performance.

**Effectiveness of each component.** Here, we study the advantages of multi-frame pre-alignment and the proposed STAM. The performance of each component is summarized in Table 2. For the baseline, we take 4 frames as input without pre-alignment and directly fuse the extracted multi-frame features together by an element-wise sum. To make comparison, we perform multi-frame alignment and then replace element-wise sum with STAM step by step. As the result shows, both the proposed modules can enhance the F1 metric, which proves the capabilities of them.

**Different modes of STAM.** We further try STAM with different modes. As introduced in Section 3.3, STAM has three modes, i.e., parallel, serial, and the mixed mode. Depending on whether CA is placed in front of SA, the serial and mixed modes have two configurations: “C-S” and “S-C”. The results of these modes are compared in Table 3. As we can see, for the proposed STAM, the mixed mode with the C-S order is able to achieve the highest F1 score.

**Comparison with other aggregation strategies.** To further verify the effectiveness of STAM, we compare it with other aggregation strategies. The results are presented in Table 4. In the first three rows, the features of multiple frames are aggregated respectively by simple element-wise summation, a double-layer ConvLSTM(Convolutional Long Short-Term Memory) [45] and a cosine-similarity-based weighted sum. The bottom three rows use attention aggregation mechanisms. ST-DANet(Spatial-Temporal Dual Attention Network) is based on DANet [46], using a pure matrix operation with softmax and two learnable weighted coefficients. ST-PSA(Spatial-Temporal Polarized Self-attention) refers to the PSA block [47], which employs convolution, pooling, and normalization operations to further enhance the representation capacity along the channel and spatial dimension. It can be discovered that using an attention mechanism could achieve higher F1 scores than other methods, among which the proposed STAM works best.

In summary, we proved the effectiveness of using multiple frames, pre-alignment, and the STAM. The ablation results also show that an input of 4 frames and using mixed mode with the C-S order for STAM can achieve the best performance. Therefore, this setting is kept for later evaluation on the Tusimple dataset.

### 4.3. Evaluation Results

#### 4.3.1. Experiments on Tusimple

We compare our method with other existing lane mark detection methods on the Tusimple dataset and the results are shown in Table 5. The highest rank is in bold and the second one is underlined. Our method is able to achieve competitive performance in terms of a high F1 value, which is very close to first place. Note that the proposed network is trained from scratch without any pre-trained models or extra training datasets.

**Figure 6:**
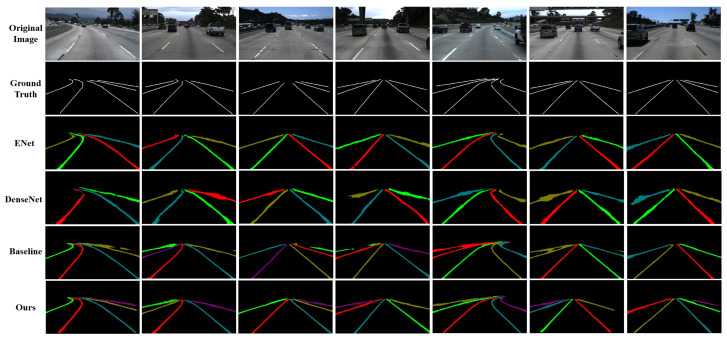
The visualization results of lane mark detection on the Tusimple dataset. We compare the proposed method with ENet [39], DenseNet [48], and our single-frame baseline. The color of lane marks is random, only for distinguishing different lane mark instances.

Figure 6 presents visual comparisons to the methods with lane instance segmentation on the Tusimple dataset. It can be observed that our method has less wrong or missing detection, reaching a better consistency with the ground-truth. Compared with those single-frame-based methods, such as ENet [39], DenseNet [48], as well as our single-frame baseline, our segmentation results have a higher localization accuracy with thinner lane contours centralizing on the true lane areas. It depresses the possibility of wrongly predicting background pixels near the ground-truth as lane mark pixels, and reduces the fuzzy adhesive region between adjacent lane marks. Besides, our method is robust to segment the entire instance of lane marks when they are occluded by vehicles.

**Figure 7:**
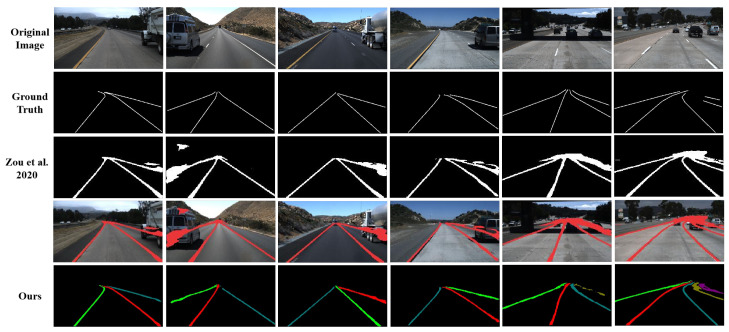
Visual comparison with the best method [28] in the Tusimple benchmark under occlusion situations.

When comparing with the best RNN-based multi-frame method [28], our method is able to overtake it in some challenging scenarios such as occluded lane marks caused by vehicles, as shown in Figure 7. To further quantitatively compare the robustness of our method and [28] under such cases, we selected 583 testing images with occlusion or shadow in Tusimple datasets for evaluation. Since the public source code of [28] does not provide instance segmentation among lanes, we added post-processing of instance segmentation above it. As shown in Table 6, although the resulting performance for total testing images is not as good as those published by the authors of [28], we only pay attention to performance degradation caused by the challenging occlusion or shadow situations. As shown in Table 6, when encounter challenging scenes, the performance of [28] decreases more than our method. The results indicate that our method does have high robustness under occlusion situations, thanks to the special design of the spatial-temporal fusion of multi-frames.

#### 4.3.2. Experiment on ApolloScape

To verify the effectiveness of the proposed method under urban environments, we further test our method in the ApolloScape dataset. As far as we know, few performances have been publicly reported on the ApolloScape Lane Segmentation dataset. Therefore, we only demonstrate the ablation results of our own method.

Firstly, we investigate the effect of fusing a different number of frames. As shown in Table 7, no matter how many frames are used, aggregating multiple frames works better than detecting lane marks in a single frame. For the ApolloScape dataset, adopting two frames can achieve optimal performance, with 3.87% and 5.02% gains on F1 scores when the threshold of IoU is 0.5 and 0.3, respectively. As the number of frames increases, the results tend to be worse. In comparison with the TuSimple dataset, a larger movement exists between the acquired neighboring images in ApolloScape, which may cause less correlations among the images.

For ApolloScape, we also evaluate the impact of each proposed component (one at a time): Alignment of multiple frames and STAM. The ablation study results are shown in Table 8. For baseline, unaligned frames are taken as input, whose features are simply aggregated by element-wise sum. To verify the effects of the proposed modules step by step, we first align the multiple frames and then insert the STAM. As we can see, no matter which IoU threshold we adopt, both multi-frame alignment and STAM are beneficial to improve performance.

**Figure 8:**
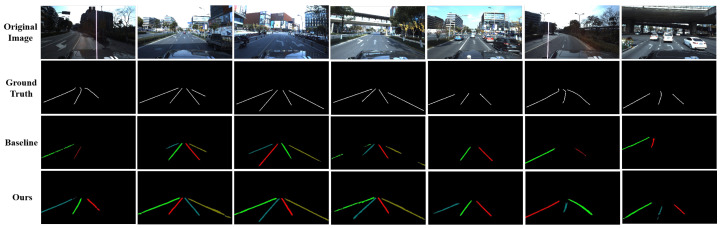
The visualization of lane mark detection results on the ApolloScape dataset. The color of lane marks is random, only for distinguishing different lane mark instances.

The visualization results on ApolloScape are demonstrated in Figure 8. Compared with the single-frame baseline, using multiple frames can better preserve the integrity and continuity of lane marks. Besides, integrated with richer information of multiple frames, our method shows strong robustness in challenging scenarios such as low illumination, vehicle occlusion, heavy shadow, and curve lanes.

## 5. Conclusions

In this work, we performed lane mark detection using multiple frames of continuous driving scenes rather than detecting the lane marks from one single image. With richer information extracted from multiple continuous images, the proposed method could achieve accurate and robust detection, despite serious vehicle occlusion, heavy shadows, and severe lane mark abrasion in some difficult conditions.

To better utilize the spatial and temporal information from multiple frames, the history frames were pre-aligned with the current key frame before entering into the encoder-decoder instance segmentation network. The sequential encoded features were attentively aggregated using the proposed STAM, followed by the two-branch decoder and post-processing to obtain the final lane mark predictions. In ablation studies, we verified the advantage of using multiple frames and the effectiveness of each proposed component. We also tried different modes of STAM and compared the STAM with other aggregating methods.

The evaluation results demonstrated that our method could achieve state-of-the-art performance, with higher F1 scores and fewer incorrect predictions than most of the single-frame methods. Furthermore, the proposed method also worked better than other multi-frame methods in some challenging scenarios, which shows stronger robustness.

## Figures and Tables

**Table 1:** Experimental results with the input of a different number of frames.

Frames	F1 (%)	Acc (%) ${}^{1}$	FP ${}^{2}$	FN ${}^{3}$
1 (w/o STAM)	93.30	95.14	0.0845	0.0488
1	94.65	95.29	0.0654	0.0413
2	96.41	96.12	0.0412	0.0306
3	96.40	96.12	0.0404	0.0315
4	**96.81**	96.20	**0.0339**	0.0299
5	96.44	**96.26**	0.0417	**0.0294**

${}^{1}$: Accuracy. ${}^{2}$: False Positive. ${}^{3}$: False Negative.

**Table 2:** Experimental results for the designed components. The number of input frames is set 4.

Alignment	STAM	F1 (%)	Acc (%) ${}^{1}$	FP ${}^{2}$	FN ${}^{3}$
		95.41	95.53	0.0528	0.0390
*√*		96.05	96.01	0.0460	0.0330
	*√*	96.22	**96.22**	0.0422	0.0333
*√*	*√*	**96.81**	96.20	**0.0339**	**0.0299**

${}^{1}$: Accuracy. ${}^{2}$: False Positive. ${}^{3}$: False Negative.

**Table 3:** Experimental results with different modes for STAM. The number of frames is 4 and multi-frame pre-alignment is used.

Modes	F1 (%)	Acc (%) ${}^{1}$	FP ${}^{2}$	FN ${}^{3}$
Parallel	96.26	**96.22**	0.0441	0.0305
Serial (C-S)	96.38	96.19	0.0389	0.0336
Serial (S-C)	96.52	96.20	0.0377	0.0318
Mixed (S-C)	96.71	96.06	0.0359	**0.0299**
Mixed (C-S)	**96.81**	96.20	**0.0339**	**0.0299**

${}^{1}$: Accuracy. ${}^{2}$: False Positive. ${}^{3}$: False Negative.

**Table 4:** Experimental results with different aggregation strategies. The number of frames is 4 and multi-frame pre-alignment is used.

Aggregation Methods	F1 (%)	Acc (%) ${}^{1}$	FP ${}^{2}$	FN ${}^{3}$
Simple add	96.05	96.01	0.0460	0.0330
ConvLSTM [45]	96.21	96.06	0.0419	0.0339
Weighted sum	96.40	**96.31**	0.0434	**0.0286**
Attention				
ST-DANet [46]	96.46	96.25	0.0398	0.0310
ST-PSA [47]	96.54	96.09	0.0357	0.0336
STAM (ours)	**96.81**	96.20	**0.0339**	0.0299

${}^{1}$: Accuracy. ${}^{2}$: False Positive. ${}^{3}$: False Negative.

**Table 5:** Comparison with other state-of-the-art methods on the Tusimple dataset.

Methods	Publications	F1 (%)	Acc (%)	FP	FN
LaneNet-HNet [17]	IV2018	94.80	96.38	0.0780	0.0244
SCNN ${}^{1}$ [18]	AAAI2018	95.97	96.53	0.0617	**0.0180**
EL-GAN ${}^{2}$ [19]	ECCVW2018	96.26	94.90	0.0412	0.0336
FastDraw [23]	CVPR2019	93.92	95.20	0.0760	0.0450
PointLaneNet [21]	IV2019	95.07	96.34	0.0467	0.0518
ENet-SAD [20]	ICCV2019	95.92	96.64	0.0602	0.0205
UFSA ${}^{3}$ [24]	ECCV2020	88.02	95.86	0.1891	0.0375
PolyLaneNet [26]	ICPR2020	90.62	93.36	0.0942	0.0933
E2E-LMD ${}^{4}$ [25]	CVPRW2020	96.58	96.22	**0.0308**	0.0376
Line-CNN [22]	TITS2020	96.79	96.87	0.0442	0.0197
Zou et al. [28]	TVT2020	**96.98**	**97.30**	0.0416	0.0186
LaneATT-R18 [9]	CVPR2021	96.71	95.57	0.0356	0.0301
LaneATT-R34 [9]	CVPR2021	96.77	95.63	0.0353	0.0292
LaneATT-R122 [9]	CVPR2021	96.06	96.10	0.0564	0.0217
Ours	/	96.81	96.20	0.0339	0.0299

${}^{1}$: Spatial-CNN. ${}^{2}$: Embedding Loss-GAN. ${}^{3}$: Ultra Fast Structure-aware. ${}^{4}$: End-to-End Lane Marker Detection.

**Table 6:** Robustness comparison with the best method [28] in the Tusimple benchmark. The less the **Δ F1** is, the higher robustness of the method. ${}^{⋆}$ means reproduced results by using its source code.

Methods	Testing Images	Acc (%) ${}^{1}$	FP ${}^{2}$	FN ${}^{3}$	F1 (%)	Δ F1 (%)
Zou et al. [28] ${}^{⋆}$	total	95.84	0.0448	0.0446	95.53	/
Zou et al. [28] ${}^{⋆}$	challenging	95.13	0.0904	0.0617	92.37	−3.16
Ours	total	96.20	0.0339	0.0299	96.81	/
Ours	challenging	95.37	0.0533	0.0483	94.92	**−1.89**

${}^{1}$: Accuracy. ${}^{2}$: False Positive. ${}^{3}$: False Negative.

**Table 7:** Experimental results of using a different number of frames.

IoU_tr	Frames	TP ${}^{1}$	FP ${}^{2}$	FN ${}^{3}$	Precision	Recall	F1	Δ F1
0.5	1	2436	**1643**	3778	0.5972	0.3920	0.4733	/
0.5	2	**2751**	1781	**3463**	**0.6070**	**0.4427**	**0.5120**	**+3.87%**
0.5	3	2647	1720	3567	0.6061	0.4260	0.5003	+2.70%
0.5	4	2601	1811	3613	0.5895	0.4186	0.4896	+1.63%
0.3	1	3303	**776**	2911	**0.8098**	0.5315	0.6418	/
0.3	2	**3804**	977	**2410**	0.7957	**0.6122**	**0.6920**	**+5.02%**
0.3	3	3676	901	2538	0.8032	0.5916	0.6813	+3.95%
0.3	4	3683	1020	2531	0.7831	0.5927	0.6747	+3.29%

${}^{1}$: Ture Positive. ${}^{2}$: False Positive. ${}^{3}$: False Negative.

**Table 8:** Effectiveness study results on ApolloScape. Here, the number of input frames is set 2.

IoU_tr	Alignment	STAM	Precision	Recall	F1	Δ F1
0.5			0.5333	0.4223	0.4714	/
0.5	*√*		0.5762	**0.4434**	0.5012	+2.98%
0.5		*√*	0.5775	0.4276	0.4914	+2.00%
0.5	*√*	*√*	**0.6070**	0.4427	**0.5120**	+4.06%
0.3			0.7755	0.5687	0.6562	/
0.3	*√*		**0.7957**	0.5803	0.6712	+1.50%
0.3		*√*	0.7831	0.5927	0.6747	+1.85%
0.3	*√*	*√*	**0.7957**	**0.6122**	**0.6920**	**+3.58%**

## Data Availability

Not applicable.

## References

[1] Hillel A.B., Lerner R., Dan L., Raz G. (2014). Recent progress in road and lane detection: A survey. Mach. Vis. Appl..

[2] Yenikaya S., Yenikaya G., Duven E. (2013). Keeping the vehicle on the road—A survey on on–road lane detection systems. ACM Comput. Surv..

[3] Kumar A.M., Simon P. (2015). Review of lane detection and tracking algorithms in advanced driver assistance system. Int. J. Comput. Sci. Inf. Technol..

[4] Sun T.Y., Tsai S.J., Chan V. Hsi color model based lane-marking detection. Proceedings of the 2006 IEEE Intelligent Transportation Systems Conference(ITSC).

[5] Li Y., Chen L., Huang H., Li X., Xu W., Liang Z., Huang J. Nighttime lane markings recognition based on canny detection and hough transform. Proceedings of the 2016 IEEE International Conference on Real-time Computing and Robotics.

[6] Li Z.Q., Ma H.M., Liu Z.Y. Road lane detection with gabor filters. Proceedings of the 2016 International Conference on Information System and Artificial Intelligence(ISAI).

[7] Liu T., Chen Z.W., Yang Y., Wu Z.H., Li H.W. Lane Detection in Low-light Conditions Using an Efficient Data Enhancement: Light Conditions Style Transfer. Proceedings of the 2020 IEEE Intelligent Vehicles Symposium (IV).

[8] Zheng T., Fang H., Zhang Y., Tang W., Cai D. Resa: Recurrent feature-shift aggregator for lane detection. Proceedings of the Thirty-Fifth AAAI Conference on Artificial Intelligence.

[9] Tabelini L., Berriel R., Paixo T.M., Badue C. Keep your eyes on the lane: Attention-guided lane detection. Proceedings of the IEEE Conference on Computer Vision and Pattern Recognition (CVPR).

[10] Lin H.Y., Dai J.M., Wu L.T., Chen L.Q. (2020). A vision-based driver assistance system with forward collision and overtaking detection. Sensors.

[11] Tu C., van Wyk B.J., Hamam Y., Djouan K., Du S. (2013). Vehicle position monitoring using Hough transform. IERI Procedia.

[12] Gackstatter C., Heinemann P., Thomas S., Klinker G. (2010). Stable road lane model based on clothoids. Advanced Microsystems for Automotive Applications.

[13] Hur J., Kang S.N., Seo S.W. Multi-lane detection in urban driving environments using conditional random fields. Proceedings of the 2013 IEEE Intelligent Vehicles Symposium (IV).

[14] Borkar A., Hayes M.H., Smith M.T. Robust lane detection and tracking with ransac and kalman filter. Proceedings of the 2009 IEEE International Conference on Image Processing (ICIP).

[15] Suttorp T., Bucher T. Learning of kalman filter parameters for lane detection. Proceedings of the 2006 IEEE Intelligent Vehicles Symposium (IV).

[16] Linarth A., Angelopoulou E. On feature templates for particle filter based lane detection. Proceedings of the 2011 IEEE Intelligent Transportation Systems Conference(ITSC).

[17] Neven D., De Brabandere B., Georgoulis S., Proesmans M., Gool L.V. Towards end-to-end lane detection: An instance segmentation approach. Proceedings of the 2018 IEEE Intelligent Vehicles Symposium (IV).

[18] Pan X., Shi J., Luo P., Wang X., Tang X. Spatial as deep: Spatial cnn for traffic scene understanding. Proceedings of the Thirty-Second AAAI Conference on Artificial Intelligence.

[19] Ghafoorian M., Nugteren C., Baka N., Booij O., Hofmann M. El-gan: Embedding loss driven generative adversarial networks for lane detection. Proceedings of the European Conference on Computer Vision (ECCV) Workshops.

[20] Hou Y., Ma Z., Liu C., Loy C.C. Learning lightweight lane detection cnns by self attention distillation. Proceedings of the IEEE International Conference on Computer Vision(ICCV).

[21] Chen Z., Liu Q., Lian C. Pointlanenet: Efficient end-to-end cnns for accurate real-time lane detection. Proceedings of the 2019 IEEE Intelligent Vehicles Symposium (IV).

[22] Li X., Li J., Hu X., Yang J. (2019). Line-CNN: End-to-End Traffic Line Detection With Line Proposal Unit. IEEE Trans. Intell. Transp. Syst..

[23] Philion J. Fastdraw: Addressing the long tail of lane detection by adapting a sequential prediction network. Proceedings of the IEEE Conference on Computer Vision and Pattern Recognition(CVPR).

[24] Qin Z., Wang H., Li X. Ultra fast structure-aware deep lane detection. Proceedings of the European Conference on Computer Vision (ECCV).

[25] Yoo S., Lee H., Myeong H., Yun S., Park H., Cho J. End-to-end lane marker detection via row-wise classification. Proceedings of the IEEE Conference on Computer Vision and Pattern Recognition (CVPR) Workshops.

[26] Tabelini L., Berriel R., Paixo T.M., Badue C., Oliveira-Santos T. Polylanenet: Lane estimation via deep polynomial regression. Proceedings of the International Conference on Pattern Recognition(ICPR).

[27] Liu R., Yuan Z., Liu T., Xiong Z. End-to-end lane shape prediction with transformers. Proceedings of the IEEE Winter Conference on Applications of Computer Vision(WACV).

[28] Zou Q., Jiang H., Dai Q., Yue Y., Chen L., Wang Q. (2020). Robust lane detection from continuous driving scenes using deep neural networks. IEEE Trans. Veh. Technol..

[29] Ketkar N. (2017). Convolutional Neural Networks. Deep Learning with Python: A Hands-on Introduction.

[30] Graves A., Mohamed A.R., Hinton G. Speech recognition with deep recurrent neural networks. Proceedings of the IEEE International Conference on Acoustics, Speech and Signal Processing (ICASSP).

[31] Zhang J., Deng T., Yan F., Liu W. (2020). Lane Detection Model Based on Spatio-Temporal Network with Double ConvGRUs. arXiv.

[32] Rublee E., Rabaud V., Konolige K., Bradski G.R. ORB: An efficient alternative to SIFT or SURF. Proceedings of the IEEE International Conference on Computer Vision(ICCV).

[33] Rosten E., Drummond T. Machine Learning for High-Speed Corner Detection. Proceedings of the European Conference on Computer Vision (ECCV).

[34] Calonder M., Lepetit V., Strecha C., Fua P. BRIEF: Binary Robust Independent Elementary Features. Proceedings of the European Conference on Computer Vision (ECCV).

[35] Fischler M.A., Bolles R.C. (1981). Random Sample Consensus: A Paradigm for Model Fitting with Applications to Image Analysis and Automated Cartography. Commun. ACM.

[36] (2017). Tusimple Benchmark. https://github.com/TuSimple/tusimple-benchmark/issues/3.

[37] Huang X., Wang P., Cheng X., Zhou D., Geng Q., Yang R. (2019). The apolloscape open dataset for autonomous driving and its application. IEEE Trans. Pattern Anal. Mach. Intell. TPAMI.

[38] Long J., Shelhamer E., Darrell T. (2015). Fully Convolutional Networks for Semantic Segmentation. IEEE Trans. Pattern Anal. Mach. Intell. TPAMI.

[39] Paszke A., Chaurasia A., Kim S., Culurciello E. (2016). ENet: A deep neural network architecture for real-time semantic segmentation. arXiv.

[40] Wang W., Shen J., Yang R., Porikli F. (2018). Saliency-Aware Video Object Segmentation. IEEE Trans. Pattern Anal. Mach. Intell. TPAMI.

[41] Ester M., Kriegel H.P., Sander J., Xu X. A density-based algorithm for discovering clusters in large spatial databases with noise. Proceedings of the Second International Conference on Knowledge Discovery and Data Mining.

[42] Abadi M., Agarwal A., Barham P., Brevdo E., Chen Z., Citro C. (2016). Tensorflow: Large-scale machine learning on heterogeneous distributed systems. arXiv.

[43] Robbins H., Monro S. (1951). A Stochastic Approximation Method. Ann. Math. Stat..

[44] Nowozin S. Optimal Decisions from Probabilistic Models: The Intersection-over-Union Case. Proceedings of the IEEE Conference on Computer Vision and Pattern Recognition(CVPR).

[45] Shi X., Chen Z., Wang H., Yeung D.Y., Wong W.K., Woo W.C. Convolutional lstm network: A machine learning approach for precipitation nowcasting. Proceedings of the Advances in Neural Information Processing Systems (NIPS).

[46] Fu J., Liu J., Tian H., Li Y., Bao Y., Fang Z. Dual Attention Network for Scene Segmentation. Proceedings of the IEEE Conference on Computer Vision and Pattern Recognition(CVPR).

[47] Liu H.J., Liu F.Q., Fan X.Y., Huang D. (2021). Polarized Self-Attention: Towards High-quality Pixel-wise Regression. arXiv.

[48] Huang G., Liu Z., Laurens V. Densely connected convolutional networks. Proceedings of the IEEE Conference on Computer Vision and Pattern Recognition(CVPR).

